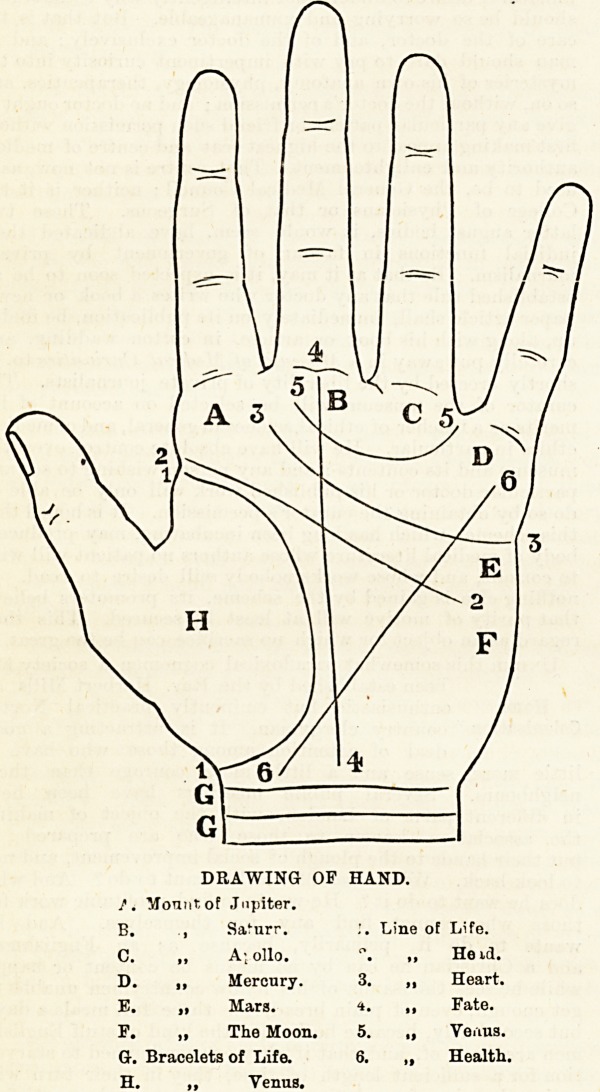# Palmistry Notes

**Published:** 1888-03-03

**Authors:** 


### AMUSEMENTS FOR CONVALESCENTS.

### PALMISTRY NOTES.

By a Lady.

(*Continued from page* 322.)

DRAWING OF HAND.

| | | | |
|---|---|---|---|
| A. Mount of Jupiter. | | | |
| B. | ,, Saturn. | 1. | Line of Life. |
| C. | ,, Apollo. | 2. | ,, Head. |
| D. | ,, Mercury. | 3. | ,, Heart. |
| E. | ,, Mars. | 4. | ,, Fate. |
| F. | ,, The Moon. | 5. | ,, Venus. |
| G. Bracelets of Life. | | 6. | ,, Health. |
| H. | ,, Venus. | | |

*Long Head Line separate from the Line of Life* gives abruptness and want of polish in speech and manner, with indifference to the feelings and susceptibilities of others. *Short separate line*, intersected with many little cross bars, shows a capricious and uncertain temper, with some vanity and jealousy. *Too long a line*, cutting the entire hand shows the evil effects of over-calculation, rendering us suspicious and avaricious. When the head line runs *down on to the Mount of the Moon* it indicates in a weak hand vacillation and want of stability. There is always a tendency to act on impulse with this formation, and we shall be imaginative and inclined to be poetical; if the Mount is much rayed there will be considerable literary power. When *the line descends very low on to the Mount* there is a danger that unbridled imagination and ungoverned impulse will lead to loss of reason. Impulse and imagination are two invaluable gifts when held in proper control, but the moment they usurp the higher function of command, the result, as we all know, is disastrous in the extreme. The moon rules the water, and when the Head Line descends low to a star on the Mount, it foreshadows death by drowning. *If the Head Line turns up to the Heart Line* (3) it shows that we allow feeling to get the better of judgment; and if the line terminates in a fork, one prong of which touches the Heart Line, a fatal infatuation has influenced our life, which, if the Line of Fate (4) cease at the Heart Line, has brought sorrow and disaster in its train.

From the matter-of-fact region of the head we turn to the consideration of the more romantic and less controllable domain over which the *Heart Line* holds its sway. Rising under Jupiter (*a*), or more generally between the fingers of Saturn (*b*) and Jupiter, the *Line of the Heart* (3) continues its course to percussion of the hand ending under the fourth finger (Mercury). If the line, as it should be, is clear and straight, without breaks or flaws, it gives us faithfulness in love and friendship, and a good disposition with feelings held well in reserve. A chained condition of the line shows caprice and uncertainty in our affections during the time that this is apparent. Breaks indicate inconstancy and broken faith; if the line is broken under Apollo, pride has been the cause; under Mercury, it has arisen from vanity and folly; while under Saturn the estrangement has come from some stroke of Fate. In this case a broken engagement has resulted not necessarily from want of affection, but from the mutual decision of those most nearly concerned, and from circumstances arising which have rendered this sad but wiser course imperative.

A fork from the line which sends one ray on to Jupiter and the other under Saturn betokens a lot of calm content resulting from gratified affection, and there is also a promise of unexpected good fortune. Too long a line of the heart indicates an excess of feeling which will interfere materially with the owner's peace of mind. If the line turn towards the Head Line under Jupiter (*a*), and is there cut by a ray, it foretells an unhappy marriage or deep heart sorrows. A falling of the Heart Line to the head at its outset shows credulity and want of business capacity.

Rays from the line show warmth of heart. A line without branches shows an unsociable and loveless disposition, and a life of toil and disappointment are foreshadowed by this condition of the line. *Absence of the Heart Line* in a good hand points to a grave fear of early and sudden decease; in a bad, deceit and faithlesnesss are indicated.

*The Line of Saturn or Fate* (4) runs up the centre of the hand, and usually ends under the second finger. When it rises thus from the wrist and ascends in a clear, strong line on to the mount of Saturn (*b*), it fortells long life and good fortune, which will enable us to overcome all obstacles. There are two other points of departure from whence the Fate Line may set out, sometimes it starts from the Line of Life (1), in which case our success in life will depend on our own efforts; or it may start from the mount of the Moon (*f*), and then we are told our good fortune will be subject to the influence and caprice of somebody else, perhaps to some sudden fancy of a rich or eccentric person, and if the Line of Fate go direct to the Heart Line (3), merging itself in the Line, it indicates a rich and fortunate marriage. It may be well to notice in passing our age as marked on this line. From the base of the line to the Head Line (2), brings us to 30 years of age; the space between the Head and Heart Lines (3) occupies a period of 15 years, thus we arrive at the age of 45; and from thence to the termination of the Line will bring us to the end of life. Sometimes our line of destiny begins indistinctly or is broken up and frayed, this tells of early difficulties; if the Line becomes clearer as it goes on, it indicates that our circumstances will improve. Upward branches show the gradual development of this happier state of things, and we shall acquire wealth and independence in later life.

(*To be continued.*)

## Figures and Tables

**Figure f1:**